# Chain mediating effects of self-efficacy and social support on the relationship between engagement in an internet-based traditional Chinese medicine nursing program and quality of life among lung cancer patients: a cross-sectional questionnaire study

**DOI:** 10.3389/fpubh.2026.1791436

**Published:** 2026-05-12

**Authors:** Xiangni Zou, Rui Guo, Yuyang Tang, Xiaofang Guan, Xinxin Zhang, Wenxia Guo

**Affiliations:** 1 Department of Nursing, The First Affiliated Hospital of Heilongjiang University of Chinese Medicine, Harbin, Heilongjiang Province, China; 2 Heilongjiang Provincial Hospital, Harbin, Heilongjiang Province, China

**Keywords:** chain mediating effect, internet-based traditional Chinese medicine nursing, lung cancer, quality of life, self-efficacy, social support

## Abstract

**Background:**

Internet-based Traditional Chinese Medicine (TCM) nursing programs may support continuous symptom management and psychosocial adaptation in lung cancer patients; however, potential pathways linking program engagement to quality of life (QOL) remain unclear.

**Objective:**

This study aimed to examine the association between engagement in an Internet-based TCM nursing program and QOL in lung cancer patients, and to test whether social support and self-efficacy sequentially mediate this relationship.

**Methods:**

We conducted a cross-sectional questionnaire study among lung cancer patients treated at the First Affiliated Hospital of Heilongjiang University of Chinese Medicine (March 2022–March 2023). QOL was assessed using the EORTC QLQ-C30, social support using the Social Support Rating Scale (SSRS), and self-efficacy using the Strategies Used by People to Promote Health (SUPPH) questionnaire. Program engagement was quantified using a prespecified implementation and evaluation score derived from platform logs and caregiver feedback. Multivariable linear regression examined associations between program engagement and QOL. Chain mediation was tested using structural equation modeling (lavaan) with bias-corrected bootstrap confidence intervals.

**Results:**

A total of 120 patients were included. In multivariable regression, higher program engagement was associated with better QOL (Model A: *β* = 1.687, *p* < 0.001). After adding social support and self-efficacy, the association attenuated but remained significant (Model B: *β* = 0.662, *p* = 0.027), suggesting potential mediation. In the chain mediation model, program engagement was positively associated with social support (*a*_1_ = 1.337, *p* < 0.001) and self-efficacy (*a*_2_ = 0.684, *p* = 0.023). Social support predicted self-efficacy (*d*_21_ = 1.032, *p* < 0.001) and QOL (*b*_1_ = 0.454, *p* = 0.002), and self-efficacy predicted QOL (*b*_2_ = 0.563, *p* < 0.001). The direct effect remained significant (*c*′ = 0.708, *p* = 0.016), consistent with partial mediation.

**Conclusion:**

Greater engagement in an Internet-based TCM nursing program was associated with better QOL among lung cancer patients, and the association was consistent with a chain pathway involving social support and self-efficacy. Prospective and randomized studies are warranted to confirm temporal ordering and causal mechanisms.

## 1 Introduction

Lung cancer remains a leading cause of cancer-related morbidity and mortality worldwide, posing a substantial public health burden (1). Due to variations in exposure to risk factors and population susceptibility, the epidemiological characteristics of lung cancer differ significantly across countries and regions. In developed nations such as the United Kingdom and the United States, lung cancer incidence and mortality have shown a declining trend (2); however, in China, the incidence remains high among males and is gradually increasing among females (3). With global population growth and aging, the disease burden attributed to lung cancer is projected to escalate further in the coming decades. It is estimated that by 2050, the number of new lung cancer cases and deaths in China will reach 1.795 million and 1.408 million, respectively, representing increases of 69.3 and 92.0% compared to 2022 (4).

In recent years, breakthroughs in diagnosis and treatment have significantly prolonged the survival of lung cancer patients, with disease-free survival ranging from 1.5 to 15.3 years for patients with stage I-IIIb disease (5, 6). Nevertheless, the overall prognosis of lung cancer patients remains unsatisfactory, with a 5-year survival rate of only 20.5% (7) and less than 10% for advanced-stage patients (8). Poor prognosis lmay substantially compromise quality of life, collectively imposing a heavy burden that substantially impairs patients’ quality of life (QOL). Compared to patients with other malignancies, lung cancer patients experience higher incidence and severity of psychological distress (9). This phenomenon is partially attributed to public cognitive biases: although lung cancer is not exclusively caused by smoking, it is widely perceived as a self-inflicted and preventable disease (10), which can lead to stigmatizing responses such as rejection, criticism, or devaluation (11), and in turn triggers negative emotions including depression, anxiety, guilt, and shame (12). Previous studies have reported that the incidence of sleep disturbance, severe fatigue, and severe depression among lung cancer patients reaches 39.5% (13, 14), 29% (7), and 13.1% (15), respectively. These symptoms often coexist and interact (16), further exacerbating QOL deterioration.

The beneficial role of social support for lung cancer patients has been well-documented (17), and supportive care needs have been clearly identified. However, the specific mechanism by which social support influences patients’ QOL remains unclear. Regardless of smoking history, lung cancer patients may experience stigma, leading them to avoid disclosing their condition to prevent discrimination. This behavior often results in missed opportunities for financial support or daily assistance (18), subsequently trapping patients in social isolation and reducing their willingness to seek help (19). Such limited social engagement directly impairs QOL. Self-efficacy, a core concept of social cognitive theory (20), has been confirmed as a key factor in symptom management and QOL improvement among cancer patients: higher self-efficacy correlates with more proactive symptom management behaviors and better QOL (21, 22).

Bandura’s Social Cognitive Theory (23) explicitly identifies four primary sources of self-efficacy beliefs: mastery experiences, vicarious experiences, verbal persuasion, and physiological/affective states. Critically, both verbal persuasion—whereby encouraging feedback and emotional support from healthcare providers, family members, and peers directly strengthen efficacy beliefs—and vicarious experience—whereby patients observe similar others successfully managing their condition and thereby develop the belief that they too can succeed—are fundamentally dependent on prior social connection and support. In other words, an individual must first have access to a supportive social network before these efficacy-enhancing mechanisms can operate. This theoretical logic establishes social support as a prerequisite condition for the cultivation of self-efficacy, supporting a temporal and causal ordering in which social support precedes self-efficacy formation. The Stress-Buffering Model (24) posits that social support attenuates the psychological impact of stressful life events by modifying cognitive appraisals of threat and perceived coping capacity. For lung cancer patients, diagnosis and ongoing treatment represent profound psychosocial stressors. Under conditions of high perceived threat and low coping resources, patients are unlikely to develop or sustain confidence in their ability to engage in self-management behaviors. Social support—particularly emotional and informational support—reduces perceived threat and enhances a sense of competence and social belonging, thereby creating the psychological conditions under which self-efficacy can develop.

In 2018, the Chinese government issued the “Guiding Opinions on Promoting the Reform and Development of Nursing Services,” emphasizing the active development of syndrome differentiation-based nursing and TCM specialty nursing, as well as the innovation of TCM nursing service models to fully leverage the role of TCM nursing in disease treatment, chronic disease prevention, rehabilitation promotion, and health care. In 2019, the “Pilot Work Plan for ‘Internet + Nursing Services’” was launched to address population aging and implement the Healthy China strategy, further expanding nursing service capacity. From the perspective of TCM nursing theory, syndrome differentiation-based care and the holistic concept emphasize coordinated regulation of physical symptoms, emotional state, sleep, diet, and rehabilitation rather than isolated management of a single symptom. In lung cancer care, TCM-specific techniques such as Baduanjin, acupoint massage, acupoint application, emotional regulation strategies, and dietary guidance may jointly contribute to symptom relief and functional recovery. Previous studies have shown that internet technology further extends these principles into home-based continuous care by enabling remote monitoring, repeated guidance, family participation, and timely communication between patients and healthcare professionals (25, 26). Guided by these policies, our hospital developed an Internet-based TCM nursing program integrating syndrome-differentiated TCM techniques with continuous online support. This program establishes a “medical staff-patient-family” collaborative care model for lung cancer patients. Although preliminary clinical observations suggested potential benefits in social support, self-efficacy, and QOL, empirical evidence and mechanistic testing of these associations are lacking.

To address this research gap, this study aimed to explore the association between the Internet-based TCM nursing program and QOL among lung cancer patients in our hospital, with a particular focus on examining the chain mediating effects of social support and self-efficacy.

## 2 Materials and methods

### 2.1 Study design and participants

We conducted a cross-sectional questionnaire study in accordance with the Declaration of Helsinki. The study protocol was reviewed and approved by the Ethics Committee of the First Affiliated Hospital of Heilongjiang University of Chinese Medicine (approval No. 2022EA011). Patients were recruited from the First Affiliated Hospital of Heilongjiang University of Chinese Medicine between March 2022 and March 2023. All participants were fully informed about the study objectives, procedures, potential risks, and benefits, and written informed consent was obtained from each participant prior to enrollment.

Patients were eligible for inclusion if they met the following criteria: pathologically confirmed primary lung malignancy; age 18 years or older; ability to use smartphones or other intelligent devices; and no cognitive impairment with the capacity to complete questionnaire surveys independently. Patients were excluded if they had comorbidities involving other severe physical diseases (such as severe cardiovascular and cerebrovascular diseases, liver or kidney failure), or had missing data on key study variables (such as QOL, social support, or self-efficacy).

A total of 223 cases were initially screened, among whom 55 were excluded due to rapid disease progression that precluded study participation, and 58 due to incomplete data. Finally, 120 patients were included in the analysis. Missing data patterns were examined, and complete case analysis was applied. All participant data were kept confidential and used solely for research purposes.

### 2.2 Study variables

#### 2.2.1 Outcome variable: quality of life

The Chinese version of the European Organization for Research and Treatment of Cancer Quality of Life Questionnaire-Core 30 (EORTC QLQ-C30 V3.0) (27, 28) was used to assess QOL. Higher functional and global health scores indicate better QOL, whereas higher symptom scores indicate greater symptom burden.

#### 2.2.2 Independent variable: implementation of “internet-based TCM”

Based on the TCM nursing foundation of our hospital and the disease characteristics of lung cancer patients, an implementation protocol and evaluation system for Internet-based TCM nursing were designed, with details shown below. The Internet-based TCM nursing program constitutes the intervention framework and study context, whereas the “program engagement score” (range: 4–19) operationalizes the degree to which each patient actually participated in and adhered to the program. Specifically, the score was derived from four prespecified indicators—completion rate of TCM operations, platform interaction frequency, health data submission rate, and family caregiver feedback rate—each reflecting a distinct dimension of patient-level engagement. This score serves as the independent variable in all analyses and represents the intensity of exposure to the program rather than a binary indicator of program participation (Tables 1, 2).

**Table 1:** Daily implementation process of “Internet-based TCM nursing”.

Time	Medical team tasks (online)	Patient tasks (home-based)	Family caregiver tasks (auxiliary)	Platform operations
7:00–7:30	• Send reminders for daily nursing tasks • Review patients’ health data from the previous day	• Perform morning Baduanjin exercise (15 min) • Measure basic blood pressure and heart rate	• Assist in adjusting patients’ exercise postures • Record measured physiological data	• Patient checks in for Baduanjin completion • Caregiver uploads basic physiological data
10:00–10:15	• Provide online Q&A for morning nursing-related questions • Push TCM emotional regulation tips and health education materials	• Perform acupoint massage (Taichong [LR3] and Neiguan [PC6], 3 min each) • Record emotional state (0–10 scale)	• Assist in locating acupoints accurately • Observe changes in patients’ emotional state	• Patient submits emotional score • Patient views emotional regulation tips
12:30–13:00	• No real-time tasks (back-end sorting of morning nursing data)	• Postprandial rest • Follow TCM dietary guidance (e.g., consume spleen-invigorating foods like Chinese yam porridge)	• Prepare meals conforming to dietary guidance • Remind patients of postprandial rest	• Caregiver uploads records of patients’ lunch diets
15:00–15:15	• Send reminders for acupoint application • Provide online guidance on application methods (for eligible patients)	• Perform acupoint application (Feishu [BL13] and Tanzhong [CV17]) • Record physical discomfort (if any)	• Assist in cleaning application sites • Secure application patches	• Patient checks in for application completion • Patient submits discomfort records
19:00–19:30	• Daily video rounds (10 min per patient, rotating schedule)• Evaluate daily nursing effectiveness • Answer questions in peer support group	• Provide feedback on daily nursing experience to the medical team • Complete VAS pain assessment (0–10 scale)	• Accompany patients during video rounds • Supplement information on patients’ daily status	• Initiate video consultation • Patient submits VAS pain score
21:00–21:30	• Send sleep guidance • Compile daily nursing records	• Evening foot soaking (15 min, with moxa leaf addition) • Record expected sleep parameters (e.g., bedtime)	• Prepare foot-soaking water • Assist patients in drying feet after soaking	• Patient submits expected sleep records • Medical team uploads daily nursing summaries

**Table 2:** Evaluation indicators and criteria of “Internet-based TCM nursing”.

Indicator	Scoring criteria (score value)	Score value	Data source
Completion rate of TCM operations	≥90% (5 points); 80–89% (4 points); 70–79% (3 points); 60–69% (2 points); <60% (1 point)	5	Check-in records on internet platform
Platform interaction rate	≥5 times/week (4 points); 3–4 times/week (3 points); 1–2 times/week (2 points); 0 times/week (1 point)	4	Platform interaction logs
Health data submission rate	≥90% (5 points); 80–89% (4 points); 70–79% (3 points); 60–69% (2 points); <60% (1 point)	5	Platform health data submission records
Family member feedback rate	≥80% (5 points); 60–79% (4 points); 40–59% (3 points); 20–39% (2 points); <20% (1 point)	5	Online feedback reports from family members

The implementation score ranged from 4 to 19, derived from platform logs (check-ins, interactions, data uploads) and caregiver feedback reports. Higher scores indicated better adherence and engagement. The scoring algorithm was prespecified and applied uniformly by trained research staff.

#### 2.2.3 Covariates

Referencing previous studies on QOL in lung cancer patients, the following covariates were selected: individual characteristics (age, gender, educational level, employment status); family-related factors (marital status, living arrangement, caregiving status); socioeconomic status (monthly household income, type of medical insurance); tumor-related factors (duration of diagnosis, pathological type, tumor stage, ongoing treatment status); and health-related factors (presence of chronic diseases).

#### 2.2.4 Mediating variables

Social support was assessed using the Social Support Rating Scale (SSRS) (29), which includes 10 items across three dimensions: subjective support (4 items), objective support (3 items), and support utilization (3 items). Items 6 and 7 were scored 0 for ‘no source’ and by the number of sources otherwise; other items were rated on a 4-point scale. The Cronbach’s alpha coefficient of the scale was 0.92, with higher scores indicating better social support.

Self-efficacy was evaluated using the Chinese version of the Strategies Used by People to Promote Health (SUPPH) questionnaire (30, 31). This scale contains 28 items across three dimensions: positive attitude (15 items), self-decision (3 items), and self-stress reduction (10 items). Using a 5-point Likert scale, total scores range from 28 to 140, with higher scores indicating stronger self-management efficacy.

### 2.3 Data collection methods

All scale assessments were completed independently by patients under the supervision of trained nurses. For patients with visual impairment or writing difficulties, a uniformly trained nurse read the questionnaire items aloud in a neutral tone and recorded the responses. All nurses involved in the assessment received the same training on questionnaire administration before the study to ensure consistency in the assessment process.

Although the key independent variable (program engagement) was derived from platform records and caregiver feedback rather than patient self-report, the mediators and outcome were measured using self-administered questionnaires. Therefore, common method bias could not be fully excluded. Harman’s single-factor test was performed to assess the potential influence of common method variance. The Harman single-factor test showed that the first unrotated factor explained 18.91% of the total variance, which was below the commonly used threshold of 40%, suggesting that severe common method bias was unlikely.

### 2.4 Statistical analysis

The sample size was determined by the number of eligible patients during the study period. We evaluated the sample adequacy against widely cited SEM-specific guidelines. Kline (32) recommends a minimum of 10 observations per estimated parameter; our model involves approximately 10 free parameters (6 structural paths plus residual variances and covariances), corresponding to a minimum recommended sample of 100. Consequently, the sample size of the present study therefore meets this requirement.

Data analysis was performed using R software version 4.3.2. The analysis consisted of three stages. First, descriptive statistical analysis was conducted, using frequency and percentage for categorical variables and mean ± standard deviation for continuous variables to clarify sample characteristics. Second, multiple linear regression analysis was performed with QOL score as the dependent variable and covariates and independent variable included to preliminarily test the association between Internet-based TCM nursing and QOL. Variance Inflation Factor (VIF) was calculated to assess multicollinearity. Third, chain mediating effect analysis was conducted using structural equation modeling implemented in the lavaan package. The model was estimated using maximum likelihood estimation, and bias-corrected bootstrapping with 5,000 resamples was employed to generate confidence intervals for the path coefficients and mediation effects. Given the cross-sectional nature of the data, the serial mediation model was used to examine whether the observed associations were statistically consistent with the hypothesized pathway rather than to establish temporal precedence or causality. Therefore, all mediation findings should be interpreted as associational rather than causal. For differential analysis of covariates, independent samples *t*-tests were used for binary variables and one-way ANOVA for categorical variables with more than two groups. Two-tailed tests were used for statistical significance, with *p* < 0.05 considered statistically significant.

## 3 Results

### 3.1 Baseline characteristics of participants

A total of 120 lung cancer patients were included in this study. The baseline characteristics of the sample are presented in Table 3.

**Table 3:** Descriptive statistics of variables (*N* = 120).

Variables	level	n (%) / Mean ± SD
*N*		120
Gender (%)	Female	29 (24.2)
	Male	91 (75.8)
Age (%)	≤60	45 (37.5)
	>60	75 (62.5)
Education (%)	Junior high and below	61 (50.8)
	Senior high	41 (34.2)
	Undergraduate and above	18 (15.0)
Employment (%)	Employed	85 (70.8)
	Retired	26 (21.7)
	Unemployed	9 (7.5)
Marital status (%)	Bereaved	4 (3.3)
	Divorced	7 (5.8)
	Married	106 (88.3)
	Unmarried	3 (2.5)
Residence (%)	City	91 (75.8)
	Country	29 (24.2)
Medical payment (%)	Insurance	90 (75.0)
	Others	28 (23.3)
	Self financing	2 (1.7)
Monthly income (%)	<3,000	29 (24.2)
	3,000–10,000	59 (49.2)
	10,000–30,000	32 (26.7)
Caregiver (%)	Children	23 (19.2)
	Others	14 (11.7)
	Parents	1 (0.8)
	Spouses	82 (68.3)
Diagnosis duration (%)	<3	7 (5.8)
	3–12	29 (24.2)
	12–36	65 (54.2)
	>36	19 (15.8)
Cancer type (%)	Adenocarcinoma	76 (63.3)
	Large cell carcinoma	3 (2.5)
	Others	14 (11.7)
	Squamous carcinoma	27 (22.5)
Cancer stage (%)	I	8 (6.7)
	II	12 (10.0)
	III	50 (41.7)
	IV	50 (41.7)
Metastasis (%)	No	49 (40.8)
	Yes	71 (59.2)
Received treatment (%)	No	50 (41.7)
	Yes	70 (58.3)
Program engagement score		10.22 ± 4.34
QLQ-C30 score		42.35 ± 20.43
SUPPH total score		77.58 ± 17.68
SUPPH level (%)	Low	21 (17.5)
	Medium	10 (8.3)
	High	89 (74.2)
SSRS total score		36.76 ± 11.30
SSRS level (%)	Low	16 (13.3)
	Medium	89 (74.2)
	High	15 (12.5)

### 3.2 Relationship between “Internet-based TCM nursing” and QOL

#### 3.2.1 Effect of covariates on QOL

Differential analysis was conducted with QOL score as the dependent variable and covariates included, with results shown in Table 4.

**Table 4:** Differential analysis of variables.

Variables	QLQ-C30 score	*t*	*p*
Gender		−0.003	0.998
Female	42.34 ± 20.19		
Male	42.35 ± 20.61		
Age (year)		7.247	<0.001
≤60	56.92 ± 15.35		
>60	33.6 ± 18		
Education level		5.931	0.005
Junior high and below	36.56 ± 21.87		
Senior high	45.29 ± 13.56		
Undergraduate and above	55.27 ± 21.94		
Employment		0.416	0.660
Employed	43.04 ± 20.57		
Retired	42.12 ± 20.41		
Unemployed	36.49 ± 20.49		
Marital status		2.025	0.216
Bereaved	31.66 ± 17.01		
Divorced	49.46 ± 6.93		
Married	42.66 ± 20.78		
Unmarried	28.96 ± 30.11		
Residence		0.448	0.655
City	42.82 ± 20.59		
Country	40.86 ± 20.2		
Medical payment		−0.529	0.598
Insurance	42.3 ± 19.77		
Others	44.62 ± 21.89		
Monthly income		8.576	0.001
<3,000	30.37 ± 23.2		
3,000–10,000	43.58 ± 19.32		
10,000–30,000	50.93 ± 14.28		
Caregiver		0.238	0.789
Children	43.33 ± 24.94		
Others	38.83 ± 19.56		
Spouses	42.65 ± 19.5		
Diagnosis duration (months)		0.467	0.706
<3	34.6 ± 18.77		
3–12	42.48 ± 21.31		
12–36	43.65 ± 20.72		
>36	40.55 ± 19.36		
Cancer type		1.438	0.235
Adenocarcinoma	42.54 ± 21.05		
Large cell carcinoma	57.55 ± 10.39		
Others	47.73 ± 18.61		
Squamous carcinoma	37.33 ± 19.54		
Cancer stage		0.854	0.467
I	41.45 ± 24.54		
II	40.19 ± 17.17		
III	39.52 ± 21.01		
IV	45.84 ± 19.94		
Metastasis		2.736	0.007
No	48.33 ± 18.88		
Yes	38.22 ± 20.56		
Received treatment		−2.745	0.007
No	36.45 ± 22.04		
Yes	46.56 ± 18.2		

Independent samples *t*-test was used for binary variables; one-way ANOVA was used for categorical variables with more than two groups.

#### 3.2.2 Correlation analysis

Correlation analysis between program engagement, social support, self-efficacy, and QOL is presented in Table 5. Results showed that both social support and self-efficacy were significantly positively correlated with QOL (*p* < 0.001), indicating that higher social support and stronger self-efficacy were associated with better QOL.

**Table 5:** Correlation analysis results.

Variables	Program engagement	SUPPH score	SSRS score	QLQ-C30 score
Program engagement	1.00	–	–	–
SUPPH score	0.53***	1.00	–	–
SSRS score	0.51***	0.75***	1.00	–
QLQ-C30 score	0.51***	0.69***	0.75***	1.00

***means *p* < 0.001.

#### 3.2.3 Multiple linear regression analysis

Multiple linear regression analysis was performed with QOL score as the dependent variable, program engagement score as the independent variable, and statistically significant covariates included. Multicollinearity diagnosis was also conducted, with results shown in Table 6.

**Table 6:** Multiple linear regression results.

Variables	Model A		Model B	
	*β*	*p*	*β*	*p*
Program engagement	1.687	<0.001	0.662	0.027
Age (year)				
≤60	R		R	
>60	−16.129	<0.001	−9.482	<0.001
Education level				
Junior high and below	R		R	
Senior high	2.276	0.446	2.74	0.265
Undergraduate and above	8.026	0.053	5.787	0.093
Monthly income				
<3,000	R		R	
3,000–10,000	7.013	0.039	3.35	0.234
10,000–30,000	12.437	0.002	4.883	0.157
Metastasis				
No	R		R	
Yes	−3.174	0.252	−3.322	0.148
Received treatment				
No	R		R	
Yes	1.856	0.509	1.8	0.437
SUPPH score			0.407	<0.001
SSRS score			0.379	0.014

Model A (only covariates and independent variable included); Model B (covariates, independent variable, and two mediating variables included).

Model A results showed that program engagement had a significant positive association with QOL in lung cancer patients (*β* = 1.687, *p* < 0.001), suggesting that higher program engagement was associated with better QOL. Results of Model B showed that after incorporating social support and self-efficacy as additional covariates, all three variables—social support, self-efficacy, and program engagement—retained significant positive associations with QOL, still had significant positive effects on QOL (all *p* < 0.05); compared with Model A, the regression coefficient of “Internet-based TCM nursing” on QOL decreased (*β* = 0.662, *p* = 0.027), suggesting that social support and self-efficacy may play mediating roles between “Internet-based TCM nursing” and QOL in lung cancer patients.

### 3.3 Chain mediating effect analysis

**Figure 1:**
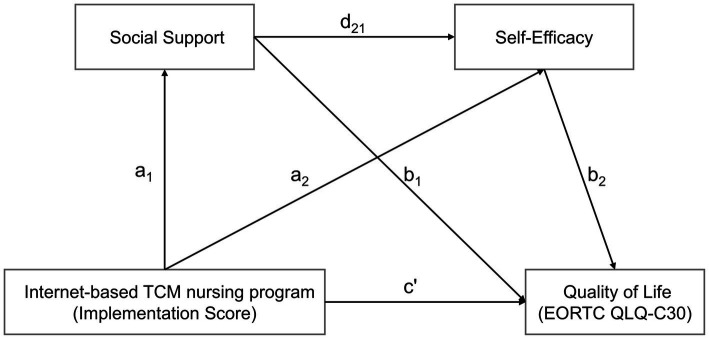
Chain mediation model of the association between the Internet-based TCM nursing program and quality of life through social support and self-efficacy.

To clarify the chain mediating roles of social support and self-efficacy between Internet-based TCM nursing program and QOL in lung cancer patients, chain mediating analysis was performed using the lavaan package. The model was estimated using maximum likelihood estimation, and bias-corrected bootstrapping with 5,000 resamples was employed to generate confidence intervals (CIs) for the path coefficients and mediation effects. Figure 1 illustrates the pathway diagram of the serial mediation analysis.

To further clarify the mediating roles of social support and self-efficacy in the association between program engagement and quality of life, a serial multiple mediation model was tested. As shown in Table 7, program engagement was positively associated with social support (*a*_1_ = 1.337, 95% CI [0.915, 1.724], *p* < 0.001) and self-efficacy (*a*_2_ = 0.684, 95% CI [0.082, 1.209], *p* = 0.018). Social support significantly predicted self-efficacy (*d*_21_ = 1.032, 95% CI [0.830, 1.254], *p* < 0.001) and quality of life (*b*_1_ = 0.454, 95% CI [0.162, 0.728], *p* = 0.002), while self-efficacy significantly predicted quality of life (*b*_2_ = 0.563, 95% CI [0.374, 0.738], *p* < 0.001). The direct effect of program engagement on quality of life remained significant after controlling for the mediators (*c*′ = 0.708, 95% CI [0.134, 1.283], *p* = 0.015), indicating partial mediation.

**Table 7:** Chain mediating analysis results.

Path/effect	Estimate [95% CI]	*p* value
Structural paths		
Program Engagement → Social Support (*a*_1_)	1.337 [0.915, 1.724]	<0.001
Program Engagement → Self-efficacy (*a*_2_)	0.684 [0.082, 1.209]	0.018
Social Support → Self-efficacy (*d*_21_)	1.032 [0.830, 1.254]	<0.001
Program Engagement → Quality of Life (*c*′)	0.708 [0.134, 1.283]	0.015
Social Support → Quality of Life (*b*_1_)	0.454 [0.162, 0.728]	0.002
Self-efficacy → Quality of Life (*b*_2_)	0.563 [0.374, 0.738]	<0.001
Indirect, total, and proportion effects		
Indirect effect via Social Support only	0.607 [0.210, 1.133]	0.009
Indirect effect via Self-efficacy only	0.385 [0.059, 0.759]	0.028
Chain indirect effect via Social Support and Self-efficacy	0.776 [0.458, 1.193]	<0.001
Total indirect effect	1.768 [1.202, 2.422]	<0.001
Total effect	2.476 [1.803, 3.090]	<0.001
Proportion mediated by Social Support only	0.245 [0.084, 0.439]	0.007
Proportion mediated by Self-efficacy only	0.155 [0.024, 0.311]	0.026
Proportion mediated by the chain pathway	0.314 [0.185, 0.488]	<0.001
Proportion of total indirect effect	0.714 [0.530, 0.939]	<0.001

In addition, the specific indirect effects were all statistically significant. The indirect effect via social support alone was 0.607 (95% CI [0.210, 1.133], *p* = 0.009), accounting for 24.5% of the total effect. The indirect effect via self-efficacy alone was 0.385 (95% CI [0.059, 0.759], *p* = 0.028), accounting for 15.5% of the total effect. The serial indirect effect via social support and self-efficacy was 0.776 (95% CI [0.458, 1.193], *p* < 0.001), accounting for 31.4% of the total effect—the largest share among the three indirect pathways. The total indirect effect was 1.768 (95% CI [1.202, 2.422], *p* < 0.001), representing 71.4% of the total effect, while the total effect was 2.476 (95% CI [1.803, 3.090], *p* < 0.001). These results indicate that social support and self-efficacy partially and jointly mediated the relationship between program engagement and quality of life, with the serial mediation pathway contributing the largest share among the three indirect pathways.

## 4 Discussion

Drawing on clinical observations, this cross-sectional study examined the mechanistic associations between Internet-based TCM nursing program engagement and QOL in lung cancer patients. This study found that greater engagement in the Internet-based TCM nursing program was associated with better QOL, and the association was consistent with a chain mediation pattern through social support and self-efficacy.

### 4.1 Direct effect of internet-based TCM nursing on QOL in lung cancer patients

This study confirmed that Internet-based TCM nursing has a significant positive direct effect on QOL in lung cancer patients, as evidenced by the significant direct effect remaining after accounting for both mediators (*c*′ = 0.708, 95% CI [0.135, 1.274], *p* = 0.016), indicating partial mediation. From the perspective of physical symptom management, TCM-specific techniques integrated in the protocol, such as Baduanjin qigong, acupoint application, and acupoint massage, can alleviate core symptoms in multiple dimensions. Within the TCM theoretical framework, lung cancer is commonly attributed to deficiency of lung Qi and obstruction of Qi and blood stasis, which give rise to the cardinal symptoms of dyspnea, fatigue, pain, and sleep disturbance. Baduanjin qigong, as a traditional health-cultivation exercise, operates through the coordinated regulation of breath, posture, and mind to replenish lung Qi, promote the circulation of Qi and blood, and restore the descending and dispersing functions of the lung. Yang et al. (33) showed that compared with traditional pulmonary rehabilitation training, Baduanjin and qigong have advantages in improving cancer-related fatigue and lung function (FEV1%)—through coordinated upper and lower limb training, they not only improve chest respiratory muscle function and exercise tolerance via upper limb movements but also regulate deep and slow breathing through lower limb movements such as squats and standing, optimizing alveolar ventilation efficiency (34) and thereby reducing dyspnea. Acupoint massage at Taichong (LR3) and Neiguan (PC6) targets the liver and pericardium meridians, respectively, with the intent of soothing liver Qi stagnation and calming the mind—mechanisms that are particularly relevant to the emotional distress and sleep disturbance frequently experienced by lung cancer patients. Additionally, a network meta-analysis explicitly recommended acupoint massage as a preferred non-pharmacological intervention for improving sleep disturbance in cancer patients (35), and improved sleep quality can further alleviate fatigue, forming a positive cycle of symptom management. Acupoint application at Feishu (BL13) and Tanzhong (CV17), guided by the TCM principle of treating Yin diseases in Yang seasons, aims to warm the lung meridian, dissipate cold pathogenic factors, and strengthen the defensive Qi, thereby alleviating dyspnea and chest distress at the level of meridian regulation. These symptom-focused components may translate into improved QOL by reducing fatigue, dyspnea, and sleep disturbance—key determinants of QOL in lung cancer.

From the perspective of psychological distress intervention, TCM nursing is grounded in the theory of emotional regulation, which recognizes the bidirectional relationship between emotional states and organ function—a concept encapsulated in the classic dictum from the Huangdi Neijing: “The seven emotions injure the internal organs.” Internet-based TCM nursing constructs a systematic psychological support system through online health education, emotional regulation guidance, and peer group interaction. This system helps patients correctly recognize the physical and psychological changes related to lung cancer, enhance confidence in treatment and rehabilitation, and reduce stigma and negative emotions (36). A randomized controlled trial (37) also confirmed that psychological education intervention for symptom management in cancer patients can not only effectively alleviate anxiety, depression, and other emotional problems but also simultaneously improve physical function, which is consistent with the direct QOL-enhancing effect of the nursing protocol in this study.

### 4.2 Mediating role of social support

Chain mediating analysis indicated that social support plays a significant mediating role between Internet-based TCM nursing and QOL in lung cancer patients, serving as an important pathway through which the nursing protocol affects QOL. Social support partially mediated the relationship between program engagement and QOL. Specifically, program engagement had a significant positive effect on social support (*a*_1_ = 1.337, 95% CI [0.911, 1.725], *p* < 0.001), which in turn significantly predicted QOL (*b*_1_ = 0.454, 95% CI [0.185, 0.749], *p* = 0.002). The significant indirect effect was confirmed as the bias-corrected bootstrap confidence interval did not include zero. From the perspective of TCM holistic theory, the human body constitutes an indivisible whole with its natural and social environment, and the maintenance of health requires not only internal Yin-Yang equilibrium but also harmonious integration within one’s relational and social world. The Internet-based TCM nursing program translates this principle into contemporary practice by constructing a “medical staff–patient–family–peer” quadrilateral interactive network, consistent with the TCM tenet of unity of the human being and its environment and the coordinated cultivation of physical and mental health. Within this framework, the collaborative participation of family caregivers in TCM-specific operations—including meridian massage, dietary preparation according to seasonal and constitutional guidance, and evening foot soaking—not only delivers technical caregiving but simultaneously reinforces the patient’s sense of emotional belonging and social connectedness through daily interpersonal interaction. In TCM emotional regulation theory, this process corresponds to the principle that “joy overcomes worry”: the positive affective experience generated through supportive social interaction promotes the free flow of Qi, disperses liver Qi stagnation, and elevates the patient’s perceived sense of being cared for and socially embedded. Firstly, Internet-based TCM nursing effectively improves patients’ social support level by constructing a “medical staff-patient-family-peer” multi-interactive network: experience sharing and emotional resonance in peer groups can reduce social isolation caused by stigma (38); professional guidance from medical staff and collaborative participation of family members not only provide practical care support but also strengthen patients’ sense of social belonging and perceived support (39). Secondly, adequate social support improves QOL through multiple mechanisms: on one hand, social support can moderate the relationship between fear of cancer recurrence and hope in lung cancer patients, thereby enhancing their hopefulness regarding rehabilitation (40); on the other hand, social support can alleviate the psychological pressure and anxiety of lung cancer caregivers, indirectly improving patients’ life experience by optimizing care quality (41).

### 4.3 Mediating role of self-efficacy

Self-efficacy has a significant mediating effect between Internet-based TCM nursing and QOL in lung cancer patients, consistent with the core tenets of social cognitive theory, with self-efficacy serving as a partial mediator. Program engagement significantly increased self-efficacy (*a*_2_ = 0.684, 95% CI [0.070, 1.219], *p* = 0.023), and self-efficacy was positively associated with QOL (*b*_2_ = 0.563, 95% CI [0.362, 0.732], *p* < 0.001). The indirect effect via self-efficacy was statistically significant. The cultivation of self-efficacy through Internet-based TCM nursing can be understood through the lens of TCM’s principle of syndrome differentiation-based nursing care, which emphasizes individualized care tailored to each patient’s specific TCM syndrome pattern—such as lung Qi deficiency or phlegm-heat obstructing the lung. This individualization is not merely a technical adaptation; it represents the activation of the patient’s own subjective agency in health self-management, consistent with the TCM philosophy of preventive cultivation, which fundamentally emphasizes the individual’s proactive responsibility for maintaining internal balance and resisting pathogenic factors. Bandura (42) proposed that self-efficacy is mainly influenced by factors such as personal success or failure experience, vicarious experience, verbal persuasion, and physiological/affective states. The Internet-based TCM nursing protocol in this study improves patients’ self-efficacy through multi-dimensional interventions: first, alleviating symptoms such as pain and insomnia through TCM-specific techniques, enabling patients to gain “success experience” in symptom management; second, providing “vicarious experience” through online case sharing and peer experience exchange; third, offering “verbal persuasion” through personalized guidance and encouragement from the medical team (43, 44).

Meanwhile, higher self-efficacy contributes to better QOL in lung cancer patients. Badana et al. (45) conducted a secondary data analysis involving 107 breast cancer patients and found that symptom occurrence affected both QOL and mental health through self-efficacy, with the direct effect of self-efficacy on wellbeing being statistically significant. Liang et al. (46) also found in 201 patients undergoing chemotherapy that symptom management self-efficacy plays a mediating role between symptom distress and QOL in all dimensions (global, symptom, function), with significant direct and indirect effects in each dimension, which is consistent with the results of this study.

The potential symptom-level mechanisms underlying these associations were not directly measured in the present study, their elucidation awaits future investigation incorporating objective clinical endpoints such as lung function indices, inflammatory biomarkers, and actigraphy-based sleep monitoring.

### 4.4 Study limitations and future directions

This study has several limitations. First, the cross-sectional design cannot confirm the causal relationship between Internet-based TCM nursing and QOL; future prospective cohort studies or randomized controlled trials are needed for further verification. Second, QOL assessment relies on patient-reported scales, which may be subject to subjective reporting bias; subsequent studies can combine objective indicators such as sleep monitoring data and inflammatory factor levels for comprehensive evaluation. Third, the current program engagement score emphasizes process-level indicators (e.g., completion rate and interaction frequency) and lacks direct measurement of effect-level indicators such as actual comprehension of intervention content and adherence quality. Future studies should develop a dual-dimensional ‘process–effect’ evaluation system—for example, incorporating structured interviews to assess patients’ mastery of TCM operational techniques and validated adherence instruments to provide a more comprehensive characterization of meaningful program participation. Fourth, although demographic and disease-related variables were controlled, unmeasured confounding factors such as cognitive function and personality traits may affect the results. Fifth, although bidirectional relationships between social support and self-efficacy cannot be ruled out, the present model prioritizes the Social Support → Self-Efficacy direction based on the theoretical frameworks described above and the logic of the program’s delivery sequence, in which the platform first establishes a multi-stakeholder support network before transmitting efficacy-enhancing information and encouragement. Nevertheless, because this was a cross-sectional study, the temporal ordering implied by the serial mediation model cannot be empirically confirmed. Alternative explanations remain possible, including reverse causation, reciprocal relations between social support and self-efficacy, and residual confounding by unmeasured psychosocial or behavioral factors. Accordingly, the present findings should be interpreted as supporting a theoretically plausible pathway rather than proving causality. Future studies employing cross-lagged panel designs or randomized controlled trials are needed to rigorously test the proposed directionality.

Future studies can be improved in the following directions: adopting randomized controlled trial design to verify the clinical efficacy of the nursing protocol; extending follow-up time to observe the long-term effect on QOL; optimizing evaluation indicators by adding objective assessment content such as TCM symptom scores and lung function; expanding sample size and conducting multi-center studies to improve the external validity of results.

## 5 Conclusion

This study demonstrates that Internet-based TCM nursing improves QOL in lung cancer patients through a direct pathway, individual mediating effects of social support and self-efficacy, and a chain mediation pathway. This nursing model organically integrates TCM-specific techniques with Internet technology, providing convenient and systematic continuous nursing services for lung cancer patients demonstrating high clinical translational value. In clinical practice, the protocol can be further optimized, and strategies for social support network construction and self-efficacy enhancement should be strengthened to better meet the health needs of lung cancer patients.

## Data Availability

The original contributions presented in the study are included in the article/supplementary material, further inquiries can be directed to the corresponding author.
